# Theoretical conceptual model as useful framework for research implementation in a Brazilian public hospital

**DOI:** 10.1186/1472-6963-14-S2-P10

**Published:** 2014-07-07

**Authors:** Mario Bracco, José Carlos Teixeira, Silvio Possa, Luiz Vicente Rizzo

**Affiliations:** 1 Hospital Israelita Albert Einstein, São Paulo, SP, Brazil; 2 Hospital Municipal Dr. Moysés Deutsch, São Paulo, SP, Brazil

## 

Translational medical science fulfils an urgent need worldwide to make knowledge from health research available as soon as possible, minimising the time between findings being made and their use in health delivery services. To plan and organize implementation of evidence-based knowledge acquisition and innovation, that could be incorporated in health services, theoretical frameworks have been developed to help its dissemination in a systematic and practical way. The Promoting Action on Research Implementation in Health Services (PARiHS) and Consolidated Framework for Implementation Research (CFIR) are conceptual models that provide variables and constructs that could be feasible for health services. The aim of this communication is to analyse PARiHS and CFIR, as guidance frameworks to research service implementation in a Brazilian public hospital.

The Hospital Dr Moysés Deutsch (HMMD) is a public facility that is managed by a public-private partnership, located in the southern zone of São Paulo City, as a reference hospital to 600,000 inhabitants. The implementation method used the PARiHS conceptual model of facilitation as a function of evidence and context, and the CFIR constructs of intervention characteristics, internal and external climate as well as organization culture. Goals, actions and indicator were developed to sensitize and stimulate the other HMMD sectors to adopt and incorporate research in their current activities, to identify researchers and evaluate the research competency among the HMMD teams. Thus, the research sector was structured to provide scientific consulting to the stakeholders’ scientific demands, and to promote and improve capacitations like scientific writing workshops; to foster, promote and implement research projects based on partnerships with other research institutes, and to submit research projects in a competitive way to get research grants from public or private agencies; to disseminate and stimulate the research culture through local scientific events and publications.

The main indicators, after two years, show an increase in the number of implemented research projects (2 to 7); the number of HMMD health professionals involved in research activities (27 to 38) and attended scientific writing workshops (4 to 17); the maintenance of papers published or submitted per year (7).

These theoretical conceptual models have an important role on guiding and monitoring HMMD research department objectives, goals, actions and indicators, improving the understanding of this research implementation process in a prospective way.

